# Estimates of Cancer Mortality Attributable to Carcinogenic Infections in Italy

**DOI:** 10.3390/ijerph17238723

**Published:** 2020-11-24

**Authors:** Pietro Ferrara, Sara Conti, Fernando Agüero, Luciana Albano, Cristina Masuet-Aumatell, Josep Maria Ramon-Torrell, Lorenzo Giovanni Mantovani

**Affiliations:** 1 Center for Public Health Research, University of Milan—Bicocca, 20900 Monza, Italy; sara.conti@unimib.it (S.C.); lorenzo.mantovani@unimib.it (L.G.M.); 2 Value-Based Healthcare Unit, IRCCS Multi Medica, 20099 Sesto San Giovanni, Italy; 3 Preventive Medicine Department, University Hospital of Bellvitge—IDIBELL, L’Hospitalet de Llobregat, 08907 Barcelona, Spain; faguero@bellvitgehospital.cat (F.A.); cmasuet@bellvitgehospital.cat (C.M.-A.); jmramon@bellvitgehospital.cat (J.M.R.-T.); 4 Clinical Science Department, University of Barcelona, L’Hospitalet de Llobregat, 08907 Barcelona, Spain; 5 Department of Experimental Medicine, University of Campania Luigi Vanvitelli, 80138 Naples, Italy; luciana.albano@unicampania.it

**Keywords:** burden of cancer mortality, cancer epidemiology, cancer etiology, cancer prevention, carcinogenic infections, modifiable risk factors

## Abstract

Several infectious agents are ascertained causes of cancer, but the burden of cancer mortality attributable to carcinogenic infections in Italy is still unknown. To tackle this issue, we calculated the rate and regional distribution of cancer deaths due to infections sustained by seven pathogens ranked as group 1 carcinogenic agents in humans by the International Agency for Research on Cancer. Population attributable fractions related to these agents were applied to annual statistics of cancer deaths coded according to the 10th International Classification of Diseases. The estimated burden of cancer mortality attributable to carcinogenic infections in Italy during the period 2011–2015 was 8.7% of all cancer deaths registered yearly, on average. Approximately 60% of deaths occurred in men, and almost the whole burden was due to four infectious agents (*Helicobacter pylori*, hepatitis C virus, high-risk human papillomavirus, and hepatitis B virus). The analysis of regional distribution showed a higher number of infection-related cancer deaths in the northern regions, where the estimates reached 30 (Liguria) and 28 (Friuli Venezia Giulia) deaths per 100,000 inhabitants in 2015. Since one-twelfth of cancer deaths were attributable to these modifiable risk factors, the implementation of appropriate prevention and treatment interventions may help to reduce the impact of these infections on cancer mortality.

## 1. Introduction

Cancer is among the leading causes of death worldwide; according to global estimates of cancer burden, around 25 million new cases, almost 10 million related deaths, and 234 million cancer-attributable disability-adjusted life years were calculated for 2017 [1].

Several infectious agents have been ascertained as causes of cancer, being responsible for at least one-sixth of all global cancers [2,3]. As easily predictable, the fraction of incident cases and deaths attributable to carcinogenic infections varies from one country to another, mainly due to different local factors, such as the geographical context or the Human Development Index [2,4]. In this frame, there is growing awareness of the importance of providing country-based estimates of the burden attributable to carcinogenic infections to provide actionable metrics for the implementation of public health interventions to reduce this preventable proportion of cancers [2,3,4,5,6].

In Italy, 371,000 new cancers were diagnosed during 2019, and currently, 3.5 million people (5.3% of the whole Italian population) live with a diagnosis of cancer. Again, around 179,000 patients died due to the consequences of these diseases, which constituted the second leading cause of death in the country after cardiovascular diseases [7]. Regarding the burden of cancers of infectious origin, the International Agency for Research on Cancer (IARC) estimated an age-standardized rate of 18.8 cases per 100,000 individuals attributable to infections [2,8], and De Flora et al. calculated that 27,000 yearly cancer cases were attributable to carcinogenic infectious agents in Italy in 2018 [5]. However, nothing has been published on the impact and trend of carcinogenic infections on cancer-related mortality in the country, except for a preliminary communication that requested more accurate and detailed analyses of this burden [6], which remain unjustifiably unknown. In fact, estimates of mortality, together with other measures of morbidity, are important elements needed to refine the understanding of whole disease burden, since incidence and prevalence alone do not offer a complete view. Furthermore, providing data on mortality and its time evolution allows for evaluation of the impact of specific implemented healthcare interventions in place [9].

Therefore, to fill this gap of knowledge, we conducted this research with the aim of estimating the burden of cancer mortality attributable to carcinogenic infections in Italy, describing the evolution on a five-year period, and estimating the regional distribution of this death toll.

## 2. Materials and Methods

We selected seven microorganisms ranked as group 1 carcinogenic agents in humans by the IARC—*Helicobacter pylori*, hepatitis B virus (HBV), hepatitis C virus (HCV), high-risk human papillomavirus (HPV) types (such as 16, 18, 31, 33, 35, 39, 45, 51, 52, 56, 58, and 59), Epstein–Barr virus (EBV), human herpesvirus type 8 (HHV-8), and human T-cell lymphotropic virus type 1 (HTLV-1)—and the global, regional, or national population attributable fractions (PAFs) and their 95% confidence intervals (95% CIs) for the listed infectious agents based on the existing body of evidence [2,4,10,11,12]. Since the data warehouse of the Italian National Institute of Statistics (ISTAT) reported data on the causes of death according to the European Short List, without the 10th International Classification of Diseases coding system (ICD-10) specification [13], we selected the World Health Organization (WHO) Cause of Death Query online (CoDQL) as our source, a web-based system for extracting trend series detailed cause of death data submitted to the WHO by its member states on an annual basis, where deaths are recorded according to the ICD-10 coding system [14]. We gathered the number of cancer-related deaths for the period 2011 to 2015 (last year available) and calculated the total number of cancer deaths by summing the death cases for each sex as reported by the WHO death statistics. The ICD-10 diagnosis codes considered in the analysis are listed in Table 1, alongside the results.

To calculate the absolute number of cancer-related deaths attributable to carcinogenic infections, we applied the evidence-based PAFs of each carcinogenetic agent to the retrieved death statistics. Following a strategy reported elsewhere [4], some analysis adjustments were performed. First, the estimation of mortality attributable to liver cancer associated with HBV and HCV infections was calculated considering their prevalence in Italy and adjusted by the relative risk of each infection [2,11]. Second, 5% of non-Hodgkin lymphoma due to *H. pylori* was assumed to be localized to the stomach [12]. Third, age-standardized relative risks for HPV-related vulvar carcinoma were considered for age ranges 15–54, 55–64, and ≥65 years [4].

To investigate the regional distribution of cancer deaths due to carcinogenic infections, the calculated national percentage of deaths attributable to infections with seven agents was applied to the regional cancer deaths statistics diffused on an annual basis by the Italian Ministry of Health, and weighted against the resident population of each Italian region according to ISTAT statistics for the same year [13,15]. To facilitate a comparison, results are presented per 100,000 inhabitants, with 95% CIs computed based on a Poisson approximation [16].

Data for the 2015 year are presented as absolute numbers, counts (percentage) with lower and upper 95% CIs (where available in literature), and mean and standard deviation (SD). Results for the previous four years are included to describe the five-year time evolution of the investigated mortality burden. Analyses were conducted with R statistical software v. 4.0.0 (The R Foundation, Vienna, Austria) [17]; the map was designed using QGIS v. 3.14.16 ‘Pi’ (OSGeo, Beaverton, OR, USA) [18].

## 3. Results

**Figure 1:**
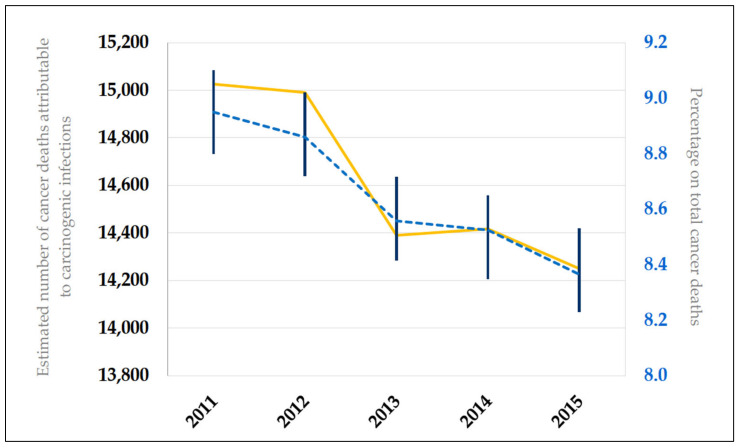
Time-series of cancer mortality burden attributable to carcinogenic infections over a 5-year period (2011–2015) in Italy. The yellow line represents the estimated numbers of cancer deaths attributable to carcinogenic infections; the dashed blue line identifies the percentage of the total cancer deaths, with 95% confidence intervals (vertical lines).

Over the five-year period, the annual number of cancer deaths attributable to infections with carcinogenic agents fell from 15,026 (2011) to 14,250 (2015), with an average of 8.7% (±0.2 SD) of all cancer-related deaths registered yearly. The time-series of this mortality burden is presented in Figure 1; a slight decrease was seen from 2011 to 2015.

For each analyzed year, roughly 60% of deaths occurred in men and almost the whole mortality burden (range 96.4–97.0%) was attributable, in equal proportions, to cancers due to four infectious agents (*H. pylori*, HCV, HPV, and HBV).

Limiting the analysis to the last available year, we found that of 14,250 estimated deaths (8.4% of the total cancer deaths; 95% CI: 8.2–8.5) due to carcinogenic infections in 2015, 58.4% (95% CI: 57.2–59.7) occurred in men. A total of 8116 deaths (57.0%; 95% CI: 55.7–58.2) were estimated to be correlated to gastric carcinoma (non-cardia and non-Hodgkin lymphoma) with *H. pylori* being the most frequent infection-related cause of cancer death in both men and women, 55.7% (95% CI: 54.1–57.3) and 58.7% (95% CI: 56.8–60.7), respectively. Liver cancer due to hepatitis viruses was responsible for 31.3% (95% CI: 30.2–32.3) of deaths, of which 3895 and 560 were attributable to HCV and HBV infections, respectively. HCV represented the second most frequent cause of carcinogenic infection death in both sexes. In women, the third leading lethal carcinogenic infections were sustained by high-risk HPV types (13.8%, 95% CI: 12.8–14.7; *n* = 816), while in men, by HBV infection (4.6%, 95% CI: 4.1–5.0; *n* = 379). Estimated deaths associated with cancer attributable to EBV were 367, mainly due to nasopharyngeal carcinoma (55.6%, 95% CI: 48.2–63.8). Table 1 offers a complete overview of 2015 estimated numbers of cancer deaths attributable to carcinogenic infections listed by infectious agents and diagnosis (according to ICD-10).

**Figure 2:**
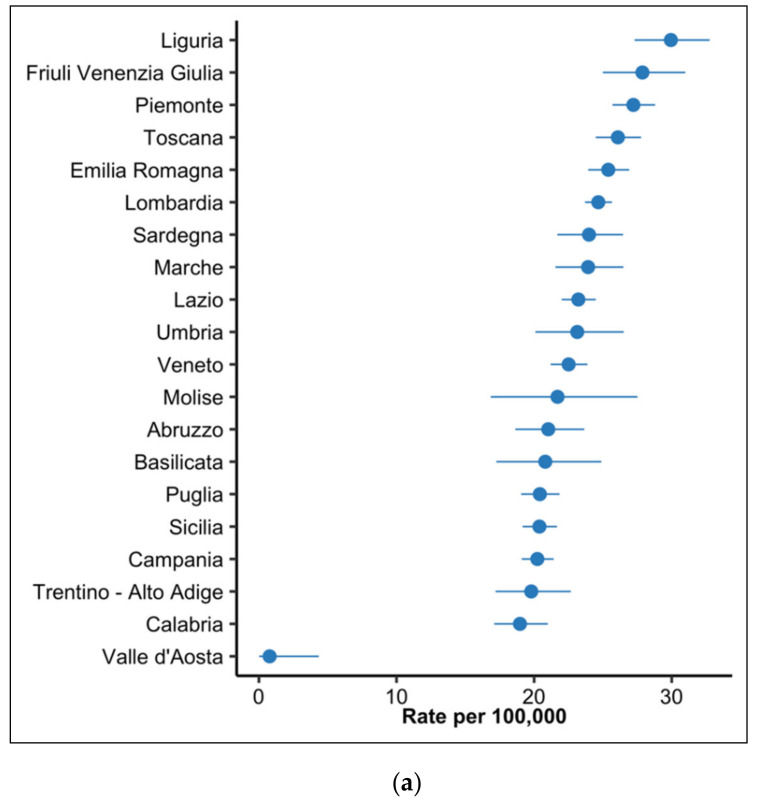

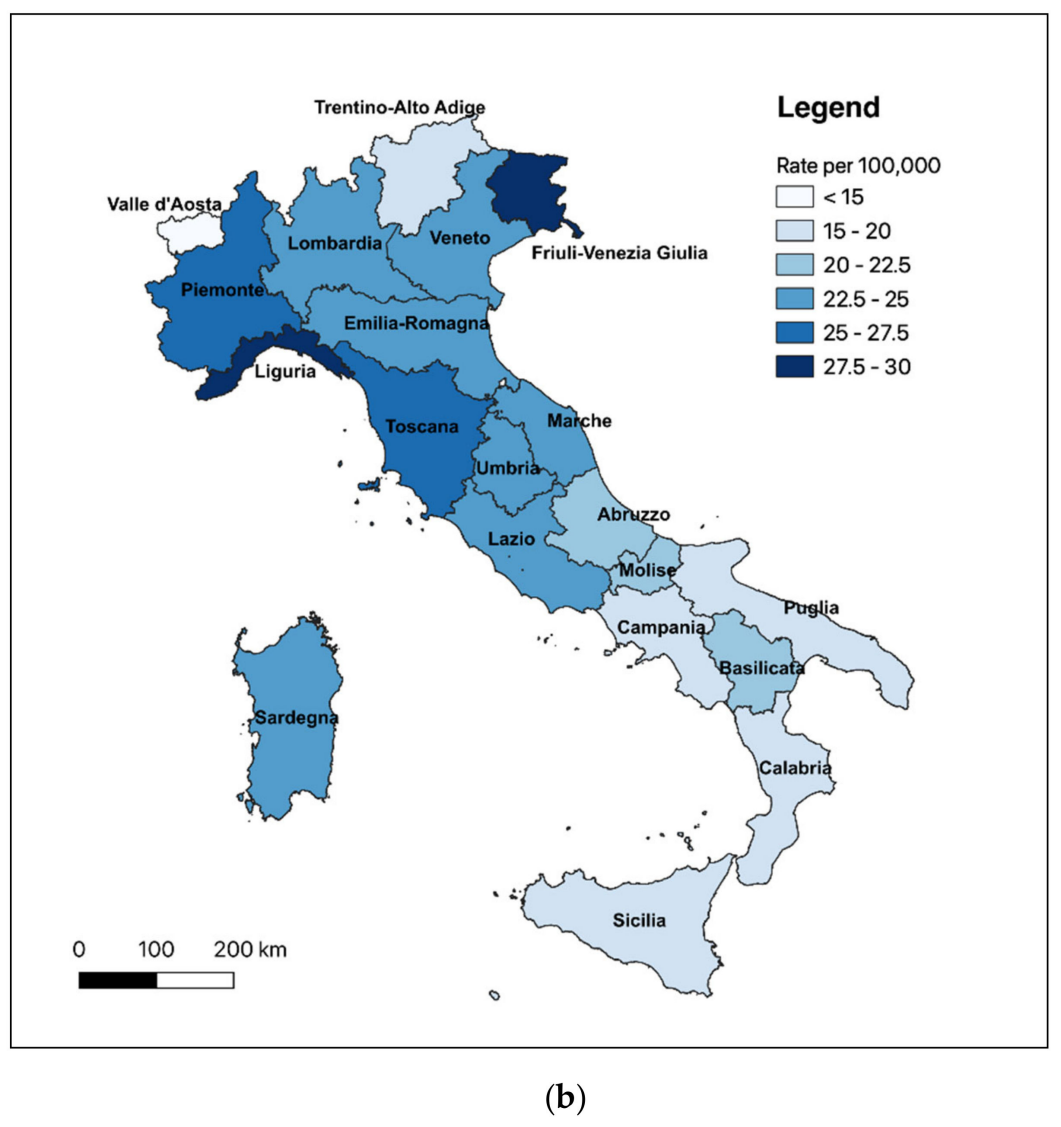
Regional estimates of cancer deaths due to carcinogenic infections per 100,000 inhabitants, Italy, 2015. (**a**) Death rates (dots) and 95% confidence intervals (lines); (**b**) Geographical distribution of death rates.

The analysis of the regional distribution of cancer mortality attributable to infections showed higher estimates of the number of infection-related cancer deaths in the northern regions (except for Valle d’Aosta); here, Liguria and Friuli Venezia Giulia regions reached, respectively, 30 (95% CI, 27–33) and 28 (95% CI, 25–31) deaths per 100,000 inhabitants. The rate gradually decreased moving toward the southern regions. The average Italian rate was 23.4 (95% CI, 23.1–23.8) deaths per 100,000 inhabitants. Figure 2 reports regional estimates and their 95% CIs.

## 4. Discussion

To the best of our knowledge, this research is the first to assess the burden, trend, and regional distribution of cancer mortality attributable to carcinogenic infections in Italy. Overall, we estimated that more than eight percent, or one out of twelve cancer deaths, were attributable to an infection sustained by microorganisms classified as group 1 carcinogenic agents in human beings by the IARC. Three cancer types, i.e., gastric, liver, and cervical cancers, represented the most frequent diagnoses, attributable to four infectious agents, namely *H. pylori*, HCV, HPV, and HBV. These results are noticeably lower than the proportion of cancer cases attributable to infections according to the last global estimates, assessed at 13% of all cancer cases [2]. However, they mirror the findings of a nation-wide study conducted in Spain in 2018, which similarly analyzed the impact of infections on cancer mortality [4].

Most of these carcinogenic agents are risk factors that can be modified through prevention measures and successful treatments, thereby avoiding a consistent number of incident cancers and deaths [2,4,6,10].

Our estimates revealed that 57.0% of all cancer deaths attributable to carcinogenic infections were related to gastric cancers attributable to *H. pylori*, which is responsible for a number of gastric diseases, including chronic inflammation and ulcers [19]. Yet, screen-and-treat strategies for *H. pylori* represent a valuable and cost-effective tool for the prevention of gastric cancer at a population level [19,20]. In Italy, local surveys estimated a 56.5% prevalence of *H. pylori* infection in the general population, and even though a regional decline has been registered, the growing antibiotic resistances are posing an important challenge for its eradication [19,21,22].

Almost one-third of the cancer mortality burden attributable to carcinogenic infections was due to HCV. A recently published survey modeled prevalence data of HCV-positive patients in the country, resulting in 443,491 cured and HCV-positive living patients and 240,043 ill patients who had yet to be treated as of 2015, with 0.18 new cases of HCV per 100,000 inhabitants [23]. Moreover, the analysis of the ITA.LI.CA (Italian Liver Cancer) register documented a 4% drop per quinquennium of HCV-related hepatocellular carcinoma (HCC) from 2000 to 2014 [11]. The recent introduction of direct-acting antivirals favorably contributed to HCV infection eradication, with valuable evidence of a lower rate of HCC development in patients who achieved sustained virologic response at 12 weeks post-treatment [24]. In this frame, access to hepatitis C treatment was expressly recommended by the WHO Thirteenth General Programme of Work (GPW13) as an indicator of country-based universal health coverage [25].

Other 560 liver cancer deaths were attributed to HBV infection. The ITA.LI.CA register estimated a decreasing prevalence of HBV infections over the last decade. The epidemiology of acute viral HBV hepatitis in Italy changed alongside the implementation of a universal HBV vaccination in 1991, mandatory for all infants and for 12-year-olds [26]. The highly effective drugs for the suppression of HBV are further contributing to reducing the cases of HBV-related liver cancers [4].

More than 1000 cancer-deaths could be attributed yearly to the high-risk HPV types. As known, this risk factor may be modified by prevention measures such as vaccination and screening programs for cervical cancer. The former has proven to provide protective benefits and to reduce cervical cancer incidence and mortality [27]. The HPV vaccination, together with cervical cancer treatment, is another indicator of effective universal health coverage according to GPW13 [25], being a valuable and effective tool for the reduction of HPV infections and anogenital warts at a population level, also protecting against cervical precancer lesions [28]. In Italy, the HPV vaccine was first recommended to all young girls aged 11 years in 2008 and then extended to boys in the twelfth year of life, men who have sex with men, and immunocompromised subjects in 2017 [29]. Longer follow-up periods are needed to show the potential effect of HPV vaccination on cancers associated with the pathogen.

We estimated that a total of 204 deaths were due to nasopharyngeal carcinoma based on the PAF for EBV. In Italy, a hospital-based study of 150 histologically-confirmed cases found a high prevalence of EBV nuclear antigen in undifferentiated nasopharyngeal carcinoma for which EBV status was known [30].

Kaposi’s sarcoma associated with HHV-8 follows the epidemiology of this herpesvirus. It is the most frequent cancer in HIV-seropositive individuals, especially those untreated [31]. In Italy, Kaposi’s sarcoma is highly prevalent in areas where HHV-8 is widespread, such as the southern regions and Sardegna Island (especially in the province of Sassari), suggesting possible predisposing conditions in populations living in those areas and a lifelong latency of HHV-8 of infection acquired during childhood, as with other herpesviruses [32,33].

HTLV-I infection in Italy appears to be rare and essentially found in migrants from endemic areas and in their sexual partners [34]. The low prevalence explains the low estimated number of deaths due to adult T-cell leukemia/lymphoma attributable to HTLV-I. Nevertheless, since one-tenth of the Italian population is constituted by migrants and refugees, potential screening interventions might be performed in the case of blood testing or donation, similar to those performed for HBV and HCV infections, to limit virus circulation [34].

We observed that the proportion of the mortality burden due to carcinogenic infections on the total cancer deaths decreased between 2011 and 2015. This reduction might be plausibly attributable to the effect of the preventive measures and treatments that have been introduced or improved over the last decade, as well as to an enhanced capacity of detecting cancer cases in early stages, which leads to a possible better chance of successful treatment [35]. The almost overlapping number of incident cancers attributable to infections between 2014 and 2018, as reported by De Flora et al. [5], would corroborate the hypothesis that the decrease in deaths due to carcinogenic infections could be related to an improvement of clinical care management of tumoral diseases rather than a diminution of cancer cases. However, more studies assessing this hypothesis over a unique time period should be implemented in the near future.

Observing the regional distribution of the rate of cancer mortality attributable to carcinogenic infections, we found interesting disparities from north to south. One main source of these differences might be regional patterns in cancer incidence [5,7,15]. For instance, it has been recognized that gastric cancer—the major contributor to the investigated mortality burden—had higher incidence and mortality in Northern Italy than in other areas of the country, probably due to a multifactorial nature (i.e., dietary pattern, *H. pylori*, alcohol and smoking habits, etc.) [36]. Again, the viral hepatitis surveillance system of the Italian National Institute of Health registers more incident cases of HCV infections in northern than in southern regions [37]. Similar patterns could likely influence the epidemiology and course of carcinogenic infections in Italy. Regional differences in cancer mortality warrant further study, also considering the age distribution of the population (i.e., the proportion of elders), the access to and compliance with prevention strategies, and other possible local factors that could have a synergistic effect on mortality in combination with infections, such as metabolic, dietary, or behavioral risk factors.

Briefly, by combining global, regional, and national PAFs for *H. pylori*, HBV, HCV, high-risk HPV types, EBV, HHV-8, and HTLV-1 with death statistics, we could estimate the burden of cancer mortality attributable to carcinogenic infections in Italy. One out of 12 cancer deaths was potentially caused by a carcinogenic agent: this class of risk factors may be preventable or treatable, and a great proportion of these deaths could be avoided with specific public health measures. Yet, it should be stressed concerning the importance of focusing on the implementation of further preventive and treatment strategies, particularly including vaccination and the promotion of healthy sexual behaviors to prevent sexually transmitted diseases, to reduce this mortality burden. Moreover, since many of the infections sustained by HTLV-1, HCV, and HBV occur in immigrants, they constitute one of the target population that calls for specific strategies for addressing this challenge [34,38].

The reduction in these infections concerns not only the possibility of avoiding a significant number of deaths but also their whole impact on health and health systems, averting disease cases and direct and social costs associated with cancer. Therefore, our findings might help primary care professionals and policymakers provide actionable metrics to adjust national and regional health care policies and enhance community health promotion for cancer prevention, screening, and care, through evidence-based information [39,40,41].

Some limitations of our research must be acknowledged. First, data were gathered in an aggregated form from publicly accessible databases, and this prevents evaluating and adjusting for potential predictors of death, such as disease history and course, tumor lethality, and survival rate. Second, the nature of the data source did not allow us to appreciate and address possible biases due to missing data or errors in recording, as well as uncertainty of sources. Third, the IARC group 1 of carcinogenic agents to humans include three more infectious pathogens, namely the parasites *Schistosoma haematobium* (associated with bladder cancer), and *Opisthorchis viverrine* and *Clonorchis sinensis* (associated both with cholangiocarcinoma); however, cancers due to these infections are predominantly seen in geographical areas where the parasites have an endemic distribution, without cases traceable in Italy [8,42,43,44].

## 5. Conclusions

In Italy, one-twelfth of cancer deaths are potentially attributable to infections due to carcinogenic agents. Many of these risk factors are both preventable and treatable through existing interventions. Data from this study may help clinicians, public health specialists, and policymakers to enhance initiatives to reduce the impact of these infections, the related mortality burden, and the associated healthcare costs.

## Figures and Tables

**Table 1:** Number of cancer deaths attributable to carcinogenic infections, by infectious agents, diagnosis (ICD-10), and sex in Italy, 2015.

	Total Deaths	PAF (95% CI) *	Total (*N*, 95% CI) ^§^	Men (*N*, 95% CI) ^§^	Women (*N*, 95% CI) ^§^
**Helicobacter pylori**					
Non-cardia malignant neoplasm of stomach (C16.1–9)	8915	89% (79–94)	7934 (7043–8380)	4538 (4028–4793)	3396 (3015–3587)
Gastric non-Hodgkin lymphoma (C82–85, C96)	245	74% (43–86)	182 (106–211)	99 (57–115)	83 (48–96)
**Hepatitis C virus**					
Liver cancer (C22)	4064	94% (92–96)	3812 (3718–3881)	2583 (2520–2630)	1229 (1199–1251)
Non-Hodgkin’s lymphoma (C82–85, C96)	4908	1.7% (1.5–2.1)	83 (74–103)	45 (40–56)	38 (34–47)
**Hepatitis B virus**					
Liver cancer (C22)	801	70% (63–76)	560 (502–610)	379 (340–413)	181 (162–197)
**Human papillomavirus (high-risk HPV types)**					
Carcinoma of the oropharynx	599	24% (17–30)	144 (102–180)	106 (75–133)	37 (27–47)
Neoplasm of base of tongue (C01)	92		22 (16–28)	17 (12–21)	5 (4–7)
Neoplasm of tonsil (C09)	186		45 (32–56)	32 (23–41)	12 (9–15)
Neoplasm of oropharynx (C10)	321		77 (55–96)	57 (40–71)	20 (14–25)
Cancer of the oral cavity	1233	4.3% (3.2–5.7)	53 (39–70)	30 (22–39)	23 (17–31)
Neoplasm of other and unspecified parts of tongue (C02)	522		22 (15–30)	13 (9–17)	10 (7–13)
Neoplasm of gum (C03)	35		2 (1–2)	1 (1–1)	1 (1–1)
Neoplasm of floor of mouth (C04)	38		2 (1–2)	1 (1–1)	1 (1–1)
Neoplasm of palate (C05)	72		3 (2–4)	2 (1–2)	1 (1–2)
Neoplasm of other and unspecified parts of mouth (C06)	566		24 (18–32)	14 (10–18)	11 (8–14)
Anal carcinoma (C21)	278	88% (85–91)	245 (236–253)	93 (90–96)	151 (146–157)
Laryngeal cancer (C32)	1480	4.6% (3.3–6.1)	68 (49–90)	61 (44–80)	7 (5–10)
Vulvar carcinoma (C51)	497	16.6% (12.5–19.8)	82 (62–98)	-	82 (62–98)
Vaginal carcinoma (C52)	93	78% (68–86)	73 (63–80)	-	73 (63–80)
Cervix uteri carcinoma (C53)	442	100%	442	-	442
Penile carcinoma (C60)	121	51% (47–55)	62 (57–67)	62 (57–67)	-
**Epstein–Barr virus**					
Nasopharyngeal carcinoma (C11)	211	96.7%	204	149	55
Hodgkin’s lymphoma (C81)	424	36% (32–39)	153 (136–165)	91 (81–99)	61 (54–66)
Burkitt’s lymphoma (C83.7)	51	20%	10	7	3
**Human herpesvirus type 8**					
Kaposi’s sarcoma (C46)	137	100%	137	76	61
**Human T-cell lymphotropic virus type 1**					
Adult T-cell leukemia/lymphoma (C91.5)	7	100%	7	3	4

* 95% CIs were not available in the literature for all the population attributable fractions considered; ^§^ Estimates for the two sexes may not add up to total estimated number of each cancer type; ICD-10, 10th International Classification of Diseases code; PAF, population attributable fraction; 95% CI, 95% confidence interval
